# Feasibility and Acceptability of Mobile Phone–Based Auto-Personalized Physical Activity Recommendations for Chronic Pain Self-Management: Pilot Study on Adults

**DOI:** 10.2196/10147

**Published:** 2018-10-26

**Authors:** Mashfiqui Rabbi, Min SH Aung, Geri Gay, M Cary Reid, Tanzeem Choudhury

**Affiliations:** 1 Department of Statistics Harvard University Cambridge, MA United States; 2 Information Science Department Cornell University Ithaca, NY United States; 3 Department of Medicine Weill Cornell Medical College New York, NY United States

**Keywords:** chronic pain, machine learning, personalization, chronic back pain, reinforcement learning

## Abstract

**Background:**

Chronic pain is a globally prevalent condition. It is closely linked with psychological well-being, and it is often concomitant with anxiety, negative affect, and in some cases even depressive disorders. In the case of musculoskeletal chronic pain, frequent physical activity is beneficial. However, reluctance to engage in physical activity is common due to negative psychological associations (eg, fear) between movement and pain. It is known that encouragement, self-efficacy, and positive beliefs are effective to bolster physical activity. However, given that the majority of time is spent away from personnel who can give such encouragement, there is a great need for an automated ubiquitous solution.

**Objective:**

MyBehaviorCBP is a mobile phone app that uses machine learning on sensor-based and self-reported physical activity data to find routine behaviors and automatically generate physical activity recommendations that are similar to existing behaviors. Since the recommendations are based on routine behavior, they are likely to be perceived as familiar and therefore likely to be actualized even in the presence of negative beliefs. In this paper, we report the preliminary efficacy of MyBehaviorCBP based on a pilot trial on individuals with chronic back pain.

**Methods:**

A 5-week pilot study was conducted on people with chronic back pain (N=10). After a week long baseline period with no recommendations, participants received generic recommendations from an expert for 2 weeks, which served as the control condition. Then, in the next 2 weeks, MyBehaviorCBP recommendations were issued. An exit survey was conducted to compare acceptance toward the different forms of recommendations and map out future improvement opportunities.

**Results:**

In all, 90% (9/10) of participants felt positive about trying the MyBehaviorCBP recommendations, and no participant found the recommendations unhelpful. Several significant differences were observed in other outcome measures. Participants found MyBehaviorCBP recommendations easier to adopt compared to the control (*β_int_*=0.42, *P*<.001) on a 5-point Likert scale. The MyBehaviorCBP recommendations were actualized more (*β_int_*=0.46, *P*<.001) with an increase in approximately 5 minutes of further walking per day (*β_int_*=4.9 minutes, *P*=.02) compared to the control. For future improvement opportunities, participants wanted push notifications and adaptation for weather, pain level, or weekend/weekday.

**Conclusions:**

In the pilot study, MyBehaviorCBP’s automated approach was found to have positive effects. Specifically, the recommendations were actualized more, and perceived to be easier to follow. To the best of our knowledge, this is the first time an automated approach has achieved preliminary success to promote physical activity in a chronic pain context. Further studies are needed to examine MyBehaviorCBP’s efficacy on a larger cohort and over a longer period of time.

## Introduction

### Background

Chronic pain is defined as pain that persists despite the resolution of injury or pathology [1] and attributed to changes in the central and peripheral nervous system resulting in amplified or uninhibited pain signals [2,3]. Persistent pain over long periods of time can affect people both physically and psychologically. Chronic pain is also closely linked with distress, affect behavior, and loss of productivity. Some studies have shown that 86% of people with chronic pain report difficulties with sleep [4], 70% have trouble concentrating [4], 44% to 51% report anxiety [5], and 88% express anger due to not seeing improvements [5]. Moreover, productivity losses can also be significant, with 4.6 hours per week rising to 5.5 hours per week lost due to chronic back pain [6]. These adverse effects can also result in other comorbidities such as depression [5,7-9]. In terms of global prevalence, the World Health Organization recognizes chronic pain as a public health problem worldwide [10] with a prevalence of 32% in low-income countries and 30% in high-income countries [11,12]. In the United States alone, 120 million adults are reported to be suffering from chronic pain [13] with related costs exceeding even that of diabetes, cancer, and heart disease [14]. Chronic pain is also related to substance abuse, with 12 million people (aged 12 years and older) reporting nonmedical use of pain medication, and overdose-related deaths are rising annually [15].

A particularly common form of chronic pain is of a musculoskeletal nature, which affects 1 in 10 adults globally. This form of chronic pain is also a leading cause of disability, with 28% reporting limitations in movement due to the condition [16]. That said, it is well understood that successful management of musculoskeletal chronic pain is achievable with regular and sustained physical activity [10]. This is due to activity having the effect of protecting against muscle weakening and inhibiting the neurophysiological mechanisms underlying the spread of pain [4]; in addition, physical activity does not have the issue of side effects that come with the consumption of pharmaceuticals [10,17]. Indeed, several recent reviews and guidelines from the US Centers for Disease Control and Prevention and UK National Institute for Health and Care Excellence have strongly encouraged clinicians to prescribe nonpharmacologic approaches that include movement-based therapies [10,18-20].

Despite the benefits of physical activity, adherence to regular and sustained physical activity is low [12,21]. This is principally due to perceived pain exacerbation from activity, which over time results in negative psychological associations (eg, fear) between movement and pain. Some of the psychological associations, typically fear and anxiety [22,23], manifest at an affective level [5] and are closely linked to more cognitive associations such as negative beliefs [5] and catastrophization [24], where subjective interpretation of pain severity may make the pain seem worse than it is [25,26]. These negative associations can lead to a reluctance to continue and even avoidance of activity and therapies [23,27] that in turn can lead to lowered self-efficacy to engage in preventative measures [24]. Lack of engagement over time can further result in weakening, disability, or even impairment in motor control where there is fproprioceptive dysfunction [28].

Low adherence to physical activity is further compounded by the need to self-manage. Typically, in day-to-day life settings, there is no care provider present to offer encouragement and guidance [12,29]. A variety of mobile phone apps have been proposed in recent years for chronic pain self-management [12,30-32]; however, the majority of mobile health (mHealth) apps to date for chronic pain do not leverage the available array of onboard sensors [33,34]. In this paper, we argue that sensor data can offer a new method that makes self-management of physical activity easier for chronic pain. Self-management becomes easier because suggestions are generated and adapted automatically from sensor data, so participants do not need to manually manage or track the suggestions they want to follow. We further reduce self-management burden by prioritizing features that make the suggestions more actionable within the psychological barriers of chronic pain [35,36]. Thus, participants do not need to use trial and error to figure out which suggestions are more or less actionable for chronic pain.

### Strategies to Address Psychological Barriers of Chronic Pain

While the utility of physical exercise for chronic pain is well known, it has also been found that introduction of new exercise tasks is more successful when small changes are made to current daily activities [12]. Other studies advocate a shift of attention strategy [37] as well as focusing on pleasurable activities [38]. These studies suggest the need to base any recommendation system on personal preferences and routine behavior. Given these lessons, we aimed to use mobile phone sensors as a natural way to acquire data on habitual behavior with respect to activity preferences. We also aimed to use this data as a basis from which new activity suggestions can be issued that are based on the contextual information taken from the tracked data. Such suggestions will likely be perceived as familiar. Also, given that the users know they have done similar activities before, this may lead to more positive beliefs and a better sense of self-efficacy. Thus, the suggestions themselves could be perceived as less effortful compared to fixed text message–based approaches, where messages are constructed before a study and the message contents do not adapt to routines or preferences of study participants [39].

### A Personalized, Self-Efficacious, and Low-Effort Suggestion Engine

We developed MyBehaviorCBP, a mobile phone app that operationalizes various strategies to address psychological barriers of chronic pain. MyBehaviorCBP uses machine learning on sensor data and self-reported physical activity logs and automatically generates physical activity recommendations based on an individual’s past behavior. This strategy of persuasion has been shown to be effective in MyBehavior, our predicating system designed for general populations [35,36]. This earlier app was designed to promote more energetic exercising and lower calorie dietary intake based on the user’s past actions. MyBehavior was shown to have affected a sustained positive behavior change within a general population in a 14-week pilot study. In this study, we repurposed this system and developed MyBehaviorCBP specifically for individuals with chronic pain. In MyBehaviorCBP, the user’s mobility state (episodes of walking or stationary state inferred from the accelerometer signals) and geolocation are passively tracked without the need for any user input. Activities that cannot be captured with sensors are entered manually. The next stage is to find recurring patterns within the physical activity data of each participant. Once recurring patterns are established, a set of new recommendations is issued based on these recurring patterns with small changes applied. We focus on these recurring patterns to generate new suggestions because participants have likely done similar actions before. Furthermore, the app uses the tracked movement data as a way to monitor which recommendations were actually preferred and executed. Subsequent recommendations are adapted based on the most acted upon previous recommendations. To the best of our knowledge, MyBehaviorCBP is the first app to promote physical activity with an automated data-driven approach in the chronic pain context.

### Objective of This Study

In this paper, we report on a formative study on the use of MyBehaviorCBP and present results from a 5-week pilot study among individuals with chronic back pain (N=10). Since the MyBehaviorCBP automated suggestion generation approach is being tested for the first time in the chronic pain context, we investigate the feasibility and acceptability of the approach before an expensive randomized controlled trial. Prior works have recommended small pilot trials (N>4) for novel mHealth apps to investigate early evidence of acceptance and use demonstrating the intervention is affecting the intended outcomes and document lessons learned, if any, for future improvements [40,41]. To this end, we (1) determined whether the MyBehaviorCBP recommendations were perceived as easy and actionable compared to randomly generated recommendations, (2) examined preliminary evidence to see whether the intentions led to an actual increase in physical activity behavior, and (3) solicited participant feedback on using the app to fine-tune future versions of the app.

## Methods

### Study Design

We conducted a 5-week within-subject study on 10 individuals with chronic back pain. The first week of the study served as a baseline period where participants familiarized with the app. No physical activity recommendation was given in the first week. The next 2 weeks were a control phase where 7 suggestions were randomly chosen every day from a pool of suggestions. This pool of suggestions was created by a fitness expert according to the US National Institutes of Health guidelines for healthy living [42,43] and these suggestions were pilot-tested in past studies [35,36]. These suggestions, however, were generic and unrelated to participants’ past behaviors. In weeks 4 and 5, the experimental phase was conducted where participants received MyBehaviorCBP-generated recommendations based on their own behaviors. The study was single-blinded in that only the experimenters were aware of when the different types of suggestions during the control and experimental phases were activated.

During each day of the control and experimental phases, participants filled out a short in-phone survey in the evening. The survey asked about the ease of following recommendations, how many recommendations they followed, and their emotional state. In addition to the daily surveys, participants completed a Web-based exit survey after the study. The exit survey asked about the helpfulness of the recommendations, what future changes they would want to see, and whether they would recommend this app to other people with chronic back pain.

### Participants

**Figure 1:**
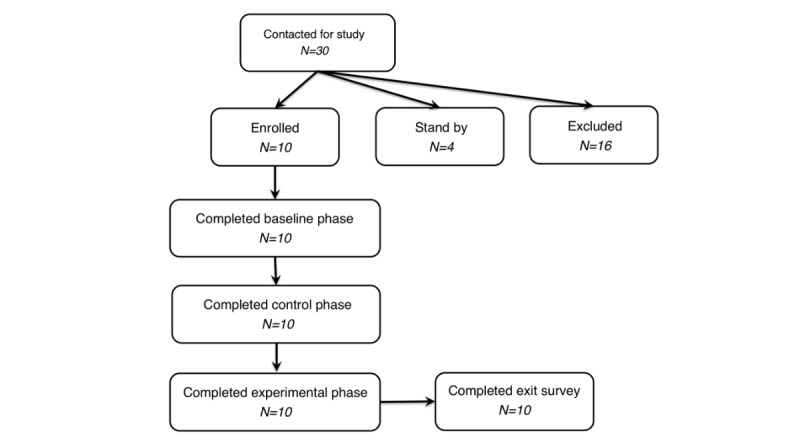
Participant flow diagram for MyBehaviorCBP pilot study.

Given the prevalence of chronic pain, invitation of the study was sent via the Wellness Center and retiree mailing lists from Cornell University. Recruitment was restricted to participants with a history of chronic back pain (≥6 months in duration) and willingness to use MyBehaviorCBP on an Android mobile phone, either their own or one provided by the study. Further inclusion criteria were having some reasonable level of outdoor movement (eg, traveling to and from work), not being significantly housebound, having a basic level of mobile phone proficiency, being between ages 18 and 65 years, and being fluent in English. Exclusion criteria, determined during an initial interview, were the need of mobility aids; having had joint replacement, arthrodesis, or limb amputation; having a learning disability; or being pregnant, but no subject fell into these categories. Eligible participants were invited for a face-to-face session where informed consent was acquired and instructions for using the app were provided (Figure 1 shows the participant flow diagram); 10 participants (3 male, 7 female, aged 31 to 60 years) were recruited, and all participants completed the study. Participants reported a range of causes for their chronic back pain, including herniated disc, rotated vertebrae, scoliosis, sciatica, and tendinitis [44] and had histories of 5 to 33 years of chronic back pain. It should be noted here that independent clinical diagnoses past that of the subjects’ own declarations about their respective causes of chronic pain were not applied. Since we are only interested in the chronic pain aspect of their condition rather the specific causes, we followed the approach of self-reporting of pain. As pain is subjective, self-report of the presence, level, and persistence of pain remains the standard assessment approach. After initial contact, some participants were further excluded as our app was limited to the Android operating system and there was a limited number of available replacement phones. Also, some potential participants were excluded because they would not be in their normal daily routines (eg, going on vacation) during the time of the user study. From the initial 30 contacted, 16 were excluded and 10 enrolled with 4 on standby.

### The MyBehaviorCBP Intervention

The MyBehaviorCBP app comprised 2 modules: routine behavior recognition module and recommendation generation module.

#### Routine Behavior Recognition Module

**Figure 2:**
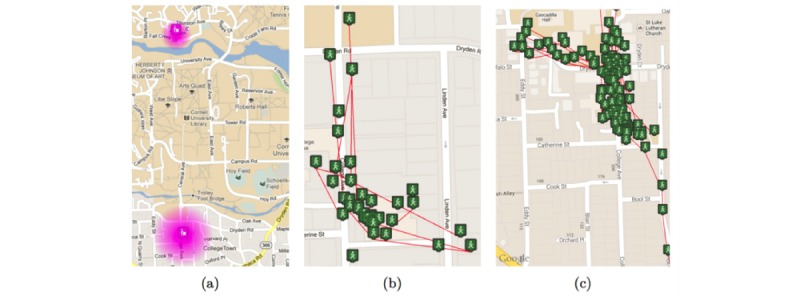
Visualization of a user’s movements over a week: (a) heatmap showing the locations where the user is stationary everyday, (b) location traces of frequent walks by the user, and (c) location traces of frequent walks by another user.

The first stage of MyBehaviorCBP is to log the physical activities of an individual with a combination of movement sensors (geolocation and accelerometer) along with manual input. Similar recurring activities are then grouped together to find routine behaviors. Specifically, activity states such as walking, running, stationary, and in-vehicle are automatically tracked using movement sensors within the phone; these activities are also tagged with the geographical location [45]. Physical activities that cannot be automatically inferred by phone sensors can be manually recorded using a drop-down menu that contains a searchable preloaded set [46]. Once an activity is logged, it is grouped together with other similar activities that have previously occurred. The method for this grouping is a data-clustering algorithm, details of which have been previously published [35,36]. The main intuition is that the same type of activities will co-occur at similar locations and are therefore assigned to the same cluster. For example, episodes of stationary state that occur in an office will have a similar GPS location and would be grouped together. Stationary episodes at a different location would be in a separate cluster from the “stationary in the office” cluster. Episodes of other mobility states are also grouped separately. For example, episodes of walking from an office to a coffee shop would show a similar trajectory of GPS locations and will be grouped together; in principle this would be a different cluster to walking from the office to a bus stand. For the manually searched and logged activities, similar exercises within the preloaded list are grouped together. Figure 2 shows a few examples of physical activity patterns extracted from 2 users.

In the interest of consistency, we will refer to each of these multifaceted clusters as a “behavior” in the remainder of this paper. Note the clustering process is determined for each participant separately without using data from other participants. Furthermore, the clustering is carried out in the phone, and no location data is exported to the cloud, minimizing privacy risks.

#### Suggestions Generation Module

Once the tracked data are grouped into different behaviors, the app then uses a sequential decision-making algorithm (multi-armed bandit or MAB [47,48]) to select and rank recommendations that are maximized to be both actionable and beneficial. In the following, we describe the main factors within the data that we consider to be important in the context of chronic pain. Subsequently, we will describe how the algorithm operationalizes these factors as part of its optimization process.

- Most frequent and repeated behaviors are prioritized. In doing this, we aim to exploit the fact that participants are familiar with these frequent behaviors and they likely have a higher level of mastery or sense of self-efficacy toward undertaking those actions [49].
- Less intensive and energetic actions are prioritized. For example, walking is prioritized over running or gym exercises. This factor is considered to promote easier or perceived as easier suggestions, which may be more compelling in situations when there is fear or anxiety of contemplating exercise [39].
- Newly generated suggestions are based on the continuation of small changes made to a user’s existing repeated behaviors. As suggested in Singh et al [12], it is attractive for those with chronic pain if less change to current behavior is needed when adopting a new therapy compared to suggestions that significantly differ from their existing routine.
- Suggestions will be uniquely contextualized to each user. Contextual information such as road or place names (Figure 2) and durational information can be added to the suggestion to further elicit a sense of familiarity [12].

In addition to the main tenets listed above, a further requirement is the need for the system to be adaptive and future proof. Since the suggestions are generated when the app is being used and data acquired, the system only has an account of the user’s past behaviors and the suggestions that have been actualized. This information is incomplete to inform what may happen in the future (eg, an ineffective suggestion from the past may become effective at a later point in time and vice versa). Thus, the system needs to have the capacity to adjust over time and adapt if necessary. Within the MAB framework, principles from the reinforcement learning (RL) branch of artificial intelligence are used, and this learning paradigm is designed to address the task of being continually adaptive. In this context, the RL agent can take a sequence of decisions in an environment to reach a predefined objective where each subsequent decision is based on the success or failure of the previous decisions.

One can consider the MyBehaviorCBP system as an RL agent as follows: let *X*=*{x_1_,x_2_,x_3_,...,x_n_}* denote the set of recommendations where each *x*_*i*_ is related to a user behavior (ie, walking to bus stop, sitting in the office). At the start of day *d*, the system chooses a subset of *X* and issues them to its user. At the end of the day, a reward score *r*_*id*_ for recommendation *x*_*i*_ is calculated according to the following quantity: *r_id_=* *m*_*id*_×*easiness* (*x*_*i*_), where *m*_*id*_ represents number of minutes a participant spent actually doing *x*_*i*_ during the day *d*; *m*_*id*_ is equal to zero if the recommendation is not adopted. The *easiness* (*x*_*i*_) function depends on how light or less vigorous the recommendation *x*_*i*_ is (eg, walking is lighter than running or gym exercise). We quantify the lightness of an activity as inverse of metabolic equivalent of task (MET) because vigorous activities tend to have higher MET values [46]. To summarize, the longer a recommendation is followed and the lower MET value it has, the higher reward it receives (eg, a longer walk would receive a higher reward than a shorter walk). But, between behaviors of the same duration but different action, eg, 30 minutes of walking and 30 minutes of gym exercise, walking would receive a higher reward because it is easier with a lower MET value.

One exception to the above equation for *r*_*id*_ is when there is a stationary behavior (eg, sitting). This is because being stationary has a low MET value, and a lot of time is generally spent in a sedentary manner. Thus, our prior formulation of *r*_*id*_ would give high reward to stationary behavior because both *m*_*id*_ and *easiness* are higher. However, rewarding more stationary behavior makes little sense, and MyBehaviorCBP’s goal is to reward more movement. Therefore, MyBehaviorCBP make the following adjustment to the reward of stationary behaviors; for each stationary behavior *x*_*j*_ϵ*X*, the reward is: *r*_*jd*_=*m*_*jd*_×*easiness* (*walking*)×3/60, where easiness of walking is the inverse of the MET value of walking activity. Defined this way, MyBehaviorCBP would suggest a small change of 3-minute walking breaks for every hour of stationary behavior.

At the end of each day *d*, 2 quantities for each *x*_*i*_ are computed: mean daily reward, *r̂*_*id*_ (*=* [*r*_*i1*_*+r*_*i2*_*+r*_*i3*_*+...+r*_*id*_]*/d*) and mean daily minutes spent *m̂*_*id*_ (*=* [*m*_*i1*_*+m*_*i2*_*+m*_*i3*_*+...+m*_*id*_]*/d*). A suggestion with high *r̂*_*id*_ means the suggestion is easy and repeated often. On day *d* +1, a new subset of recommendations is chosen with the following composition: 80% with the highest *r̂*_*id*_ and 20% randomly chosen from the remaining; moreover, the total *m̂*_*id*_ for the selected recommendations cannot exceed 60 minutes. In this setup, MyBehaviorCBP ensures 80% of the recommendations are easy and have been frequently followed before. For the remaining 20%, we allow for exploration of other recommendations to see if they get adopted. Finally, the limit of 60 minutes is set to aim for a predefined duration of exercise. This is done since the therapeutic objective here is not necessarily to encourage as much activity as possible but to regularly adhere to some predefined target. To operationalize this, we use a MAB variant called the multi-armed bandit with knapsack [50] using an *ε*-greedy strategy [48]. This would encourage the issuance of easier-to-do recommendations that simply target reaching 60 minutes rather than having no upper bound.

**Figure 3:**
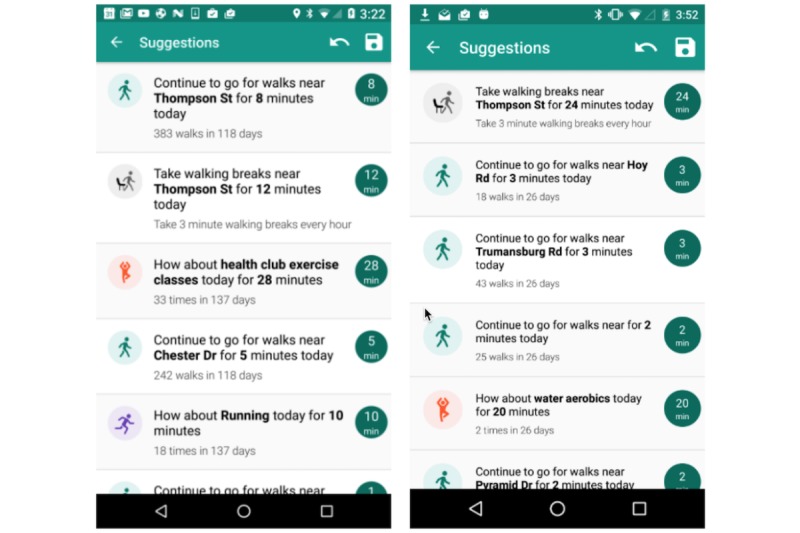
MyBehaviorCBP’s personalized suggestions for 2 users.

Figure 3 shows examples of MyBehaviorCBP suggestions for 2 different users. The screenshots are distinct and show MyBehaviorCBP’s capability to personalize to different users. Short encouraging texts are also added to the recommendations, such as (1) continuing existing walking behaviors, (2) taking short walking breaks as a small change to stationary behaviors, or (3) encouraging to continue other exercises. Along with this text, the app shows how many minutes are achievable (equal to *m̂*_*id*_) and should be done for each of the suggestions.

### Measures

The MyBehaviorCBP system intends to encourage more physical activity over a sustained period of time. However, given the early stage of the technology, a 5-week pilot study was conducted. The goal of the pilot was to investigate the feasibility of MyBehaviorCBP, which was measured by 3 factors: use, acceptability, and early efficacy. In addition, we report lessons learned for future improvements [40,41,51]. These factors are measured with a combination of phone logs, daily evening surveys during the study, and an exit survey (see Table 1 for measured outcomes). Phone logs contain passively collected records of whether MyBehaviorCBP was opened and the type of physical activity (ie, walking, stationary) for every minute during the study. The daily evening surveys were done in the phone each day of the 5-week study; in these daily evening surveys, we asked about relative ease of the recommendations received, how many recommendations they followed, and their emotional state during the day. After the 5-week study, an exit survey was conducted on the Web that asked about the helpfulness of the recommendations, what future changes they would want to see, and whether they would recommend this app to other people with chronic back pain.

Use was measured by how frequently study participants opened the app, recorded from the phone log. Acceptability, a more complex quantity, was measured by traingulating a variety of self-reports that focused on intention and behavior toward the recommendations. We specifically measured perceived easiness, which indicates the actionability of the recommendations [39,49], intention, behavior, and helpfulness. Early efficacy is another complex quantity we measured with several proximal measures that can lead to reduction of pain in the long term [52]. Finally, open-ended questions were used to pin down which features participants found useful and what features they saw as missing; this data will be used to map out future refinements of MyBehaviorCBP.

### Analysis Plan

Number of times the app is accessed was analyzed using the simple descriptive statistics of mean and standard deviation. The acceptability and early efficacy outcomes are less straight forward to analyze because data points from the same subject being likely correlated and different subjects having different baseline conditions at the start of the study (eg, different levels of physical activity and type of chronic back pain) [53,54]. Therefore, we apply linear mixed-effect models to adjust for repeated measures and intersubject variability [53].

**Table 1:** Different outcome measures captured in the MyBehaviorCBP pilot study and their purposes.

Data collection methods and description of outcome measure			Purpose of outcome measure
**App use**			
	Record of how many times the app is opened	Use	
**Physical activity log**			
	Number of minutes spent walking per day	Early efficacy	
	Number of minutes spent in nonwalking exercises per day	Early efficacy	
**Daily evening survey**			
	Perceived easiness: How easy did today’s suggestions seem after reading them? (Likert scale: 1=I could never do these suggestions to 7=I could always do these suggestions)	Acceptability	
	Intention: How many suggestions did you want to follow today? (integer value between 0 and 7)	Acceptability	
	Behavior: How many suggestions did you follow today? (integer value between 0 and 7)	Acceptability	
	Pain level: Please indicate your pain level today. (Likert scale: 0=no pain to 10=extreme pain)	Early efficacy	
**Exit survey**			
	Did receiving suggestions from your phone help you to be more active? (multiple choice: not helpful, somewhat helpful, very helpful)	Acceptability	
	How likely are you to recommend this app to another person with back pain? (multiple choice: not likely, somewhat likely, very likely)	Acceptability	
	What changes do you think could be made to the app that would make it more effective in helping you be more active? (open-ended)	Future improvement	

The type of intervention is considered as a fixed effect, and we coded the intervention type as 0 and 1 for control (ie, the static suggestions generated by experts) and experimental phases (ie, MyBehaviorCBP suggestions), respectively. Coded this way, the intervention coefficient would represent the relative improvement of the outcome measure of MyBehaviorCBP over the control. When we included time (as day within the study) as a fixed effect, it was found to be not significant. Also, we tested the study participant identity as a random effect and found it to be significant in likelihood ratio tests (*P*<.01) for all outcome measures [53], which means significant interpersonal variability exists and a mixed-effect model is necessary. The parameter estimation for all models was generated using maximum likelihood estimation [53]. In addition, we computed effect size measures by dividing the mean difference of an outcome measure between the control and experiment phases with pooled standard deviation [55]. Finally, the open-ended questions in the exit survey are broken down into themes using thematic analysis [56].

## Results

### Use

**Figure 4:**
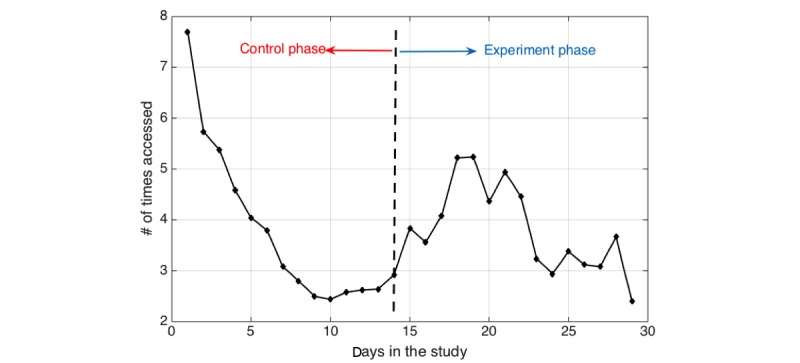
Number of times a day MyBehaviorCBP app was accessed.

Over the 5-week study with 10 participants, the mean number of times the MyBehaviorCBP app was opened is 106.9 during the control and experiment phases (*μ*=106.9, σ=56.9, *q*_25_=76.1, *q*_50_=89.6, *q*_75_=105.5), which is 3.2 times on average per day. Figure 4 shows the average number of times a participant opened the MyBehaviorCBP app over time. For both control (ie, static suggestions) and experiment phases (ie, MyBehaviorCBP-generated suggestions), there is an initial period of high use but over time the use decreased.

### Acceptability of the Suggestions

In the exit survey, the participants reacted positively about MyBehaviorCBP recommendations, with 2 of 10 participants finding MyBehaviorCBP recommendations very helpful and 8 of 10 finding MyBehaviorCBP recommendations somewhat helpful. No participant reported the recommendations unhelpful. All participants (10/10) reported that they would likely recommend the app to other people with chronic back pain.

**Figure 5:**
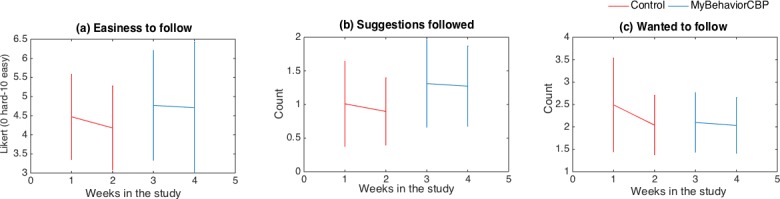
Mean and standard deviations of acceptability measures.

The acceptability of MyBehaviorCBP was also measured using (1) self-reported rating of easiness of the recommendations, (2) how many recommendations the participants wanted to follow, and (3) how many recommendations the participants actually followed. The results of the statistical analysis are reported in Table 2, and Figure 5 shows the mean and standard deviations. In the interest of convenience, only important statistics are shown in Table 2. The intervention coefficients (*β*_*int*_) are reported along with *P* values (*P*_*int*_) and 95% confidence intervals. We observe there were significant changes of *β*_*int*_ for number of recommendations followed (*β*_*int*_=0.46, *P*<.001). In real terms, participants within the experimental phase adopted 1 extra recommendation every 2 days. On the other hand, participants wanted to follow the control phase recommendations more than the experimental phase (*β*_*int*_=–0.2, *P*=.02), which means control group recommendations were effective to increase intention but not for actualizing a recommendation. Regarding easiness, participants perceived the MyBehaviorCBP recommendations to be easier than the control, which means they were perceived as low-burden (*β*_*int*_=0.42, *P*<.001). This is important in the chronic pain population, which is often reluctant to be physically active.

**Figure 6:**
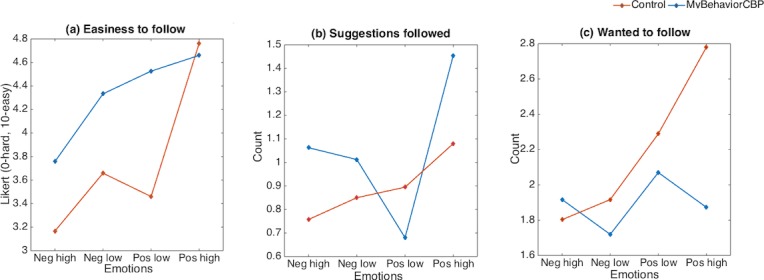
Means of several outcome measures for different emotional states.

The number of self-reported recommendations followed and wanted to follow, however, had important differences for different emotional states in the day. Figure 6 shows the means for several outcomes under different emotional states captured through the PAM [57]. Generally, participants adopted or wanted to adopt more recommendations during positive emotional states. However, during high positive emotional states, generic recommendations were perceived as easy and participants wanted to follow them more, but the MyBehaviorCBP recommendations were adopted more. During negative emotional states, the number of recommendations participants wanted to follow and actually followed are both higher than the control group recommendations.

**Table 2:** Summary of differences between control and MyBehaviorCBP as collected from survey and physical activity logs.

Outcome measure	*β_int_*	*P_int_*	95% CI_*int*_	*d*	–2logL	AIC^a^	BIC^b^	LR^c^
How easy were the suggestions	0.42	<.005	0.2 to 0.6	0.25	817.5	879	894.6	0.009
# of suggestions followed	0.46	<.005	0.2 to 0.7	0.11	4795	4809	4839	0.01
# of suggestions wanted to follow	–0.2	.02	–0.5 to –0.1	–0.2	4795	4809	4839	0.002
Walked (minutes/day)	4.9	.02	0.8 to 8.9	0.31	2123	2131	2144	0.009
Exercised (minutes/day)	9.5	.31	–6.3 to 21.8	0.03	2986	2993	3008	0.01
Pain level	–0.19	.24	–0.5 to 0.14	0.17	1160	1168	1183	0.001

^a^AIC: Akaike information criterion.

^b^BIC: Bayesian information criterion.

^c^LR: likelihood ratio test between the fitted models compared to unconditional mean models [35,53].

**Figure 7:**
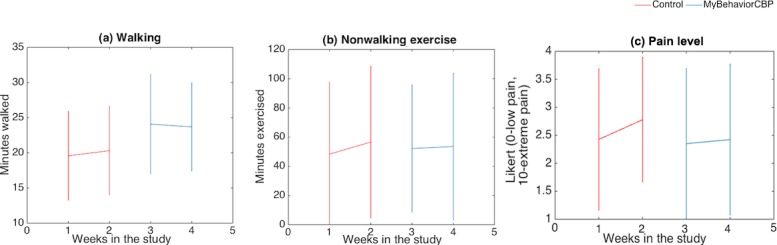
Mean several outcome measures for different preliminary efficacy outcomes.

### Preliminary Efficacy on Increasing Physical Activity and Reducing Pain

From Table 2 and Figure 7, the intervention coefficient for daily walked minutes (*β*_*int*_=4.9, *P*=.02) indicates that participants walked for a further 4.9 minutes per day compared to the control phase. *β*_*int*_ for nonwalking exercise (*β*_*int*_=9.5, *P*=.31) indicated that participants in the experimental phase exercised for a further 9.5 minutes, but the difference was not significant. Regarding pain level, there was a small nonsignificant 0.19 unit of reduction of pain (*β*_*int*_=–0.19, *P*=.24).

### Qualitative Feedback and Lessons Learned

Participants provided qualitative feedback in the exit survey, which provided further insights about the quantitative results and gave directions for future changes. For instance, when we asked participants to compare control group recommendations with MyBehaviorCBP, participants reported that they liked the personalization of MyBehaviorCBP. They also mentioned MyBehaviorCBP recommendations were more actionable and easier, and they were more likely to succeed if they tried the recommendations.

I really liked the personalization. I thought it was a nice touch. Suggestions were more specific and tailored, which for me made them more relevant and likely for me to use them.

P1

...most of the suggestions were fairly easy; at least the ones that involved walking.

P3

Because the suggestions of MyBehaviorCBP were based on my own chosen activities, I was much more likely to follow them.

P4

I liked them more because it seemed more likely that I could do them—I was more likely, in my mind, to succeed.

P6

Again, because the suggestions were based on my activities, they felt more feasible. I didn't have to take the extra step of thinking about how I might get the right tools (eg, bike) or where I can do the suggested exercise.

P9

Other than changing the sitting behavior, I liked the fact that they seemed more do-able.

P10

In addition, some participants liked the specificity of the recommendations and how they could be carried out in a smaller piecewise manner.

...they were location specific, smaller chunks of time.

P3

...more detailed explanations/suggestions, based on past exercises logged, and having the location helped, too!

P8

Regarding the control phase suggestions, some participants struggled with their nonpersonalized nature and how they needed to plan ahead to execute them.

...which I wanted to do the longer suggestions in version 2 [ie, control phase], unless I scheduled or planned it, I couldn't do most of them.

P8

...I received the suggestion to ride a bike, but that's currently simply not possible, logistically.

P1

However, one participant did not like MyBehaviorCBP recommendations and wanted more variety.

There was very little variation in the suggestions during the final 2 weeks—almost everyday was walk slightly farther and play tennis for a few minutes more... In the 2 to 3 weeks, there was a greater variety of things to try and I tried a few novel suggestions.

P2

Participants also asked for the following features: (1) a reminder system to plan in the morning and notifications in the moment, (2) adapt suggestions based on weather or weekend/weekday, and (3) better insight to relate high pain days and activity level, etc.

It would be helpful to have reminders and suggestions pop up in the morning or at other chosen times. This could be optional and set by the user.

P1

Maybe adding an alarm or something, to say “here, you should go do this thing now.” I think if I had something bugging me to get up and take a short walk, for example, I would be more likely to do it than just looking at a list of things I might do.

P3

If it could ask me to rank the things I enjoy doing and then download weather data for the following days. This could suggest times when I have performed these tasks in the past and also match it with weather predictions. “You played tennis last Tuesday in the afternoon for 90 minutes. How about from 2 to 4 today when the weather will be clear and 85.”

P7

Maybe a tally at the end of each week regarding days unable to exercise, based on back pain.

P10

Finally, one participant wanted to use the app even after the study, and mentioned the following:

I liked this app and look forward to possibly using it permanently in the future.

P9

## Discussion

### Principal Findings

To the best of our knowledge, MyBehaviorCBP is the first mobile app to provide automatically generated data driven physical activity recommendations in the chronic pain context. We conducted a pilot study to examine the feasibility of the approach. In the study, we found participants used the MyBehaviorCBP app 1 or more times a day. Furthermore, we observed early indication of acceptance and efficacy in both the qualitative and quantitative data. For instance, in the daily surveys, participants perceived the tracked data-based recommendations to be easier to follow. In the qualitative feedback on the exit survey, participants were positive to successfully complete MyBehaviorCBP recommendations. This means participants likely had a greater sense of self-efficacy toward MyBehaviorCBP-generated suggestions. According to protection motivation theory, higher self-efficacy may cause the recommendations to be carried out despite the presence of fear in chronic pain [58]. Indeed, empirically the participants also acted on the MyBehaviorCBP suggestions according to the automated walking inference. This increase gives early evidence that the perceived easiness may have transferred to actual behavior. However, the change in nonwalking exercise was not significant. This may have happened because it is hard to ascertain regularity in exercising within a 2-week period [35]. Furthermore, there was a small reduction of reported pain, which was not significant. This can be a type II error, and the small-scale study may not be sufficient to reject the system’s efficacy [54].

### Scopes for Future Improvement

From Figure 4, we observed that the number of times participants opened the app decreased over time. This may mean engagement decreased over time. Fortunately, participants also gave us valuable feedback to improve future versions of the app, which may increase engagement. For instance, several of our participants asked for notifications when the context was appropriate for a recommendation. Indeed, Fogg [39] argues that a trigger or notification may be necessary even when the suggested action can be executed with less effort. However, providing just-in-time notifications has particular technical barriers since it requires constant monitoring to detect the right context without draining the phone battery. In addition, the notifications can interrupt the participants’ daily workflow, and it is unclear when the right time to provide an intervention would be. Therefore, future research may focus on just-in-time interventions that are acceptable to participants and more battery efficient.

### Relationship With Earlier MyBehavior Work

The current MyBehaviorCBP system is a variant of a prior system, MyBehavior [36]. MyBehaviorCBP borrows several ideas from MyBehavior, such as clustering routine activities and the MAB algorithm. However, there are a few key differences between MyBehavior and MyBehaviorCBP. MyBehavior was designed for a weight loss population with no chronic pain, and the objective function of the MAB was to maximize calorie expenditure. On the other hand, MyBehaviorCBP is designed for people with chronic pain, and the goal of the MAB is to maximize number of minutes performing low-effort exercises. As a result, between walking and gym exercise, MyBehaviorCBP would prioritize walking since walking is less effortful, but MyBehavior would prioritize gym exercises since gym exercises give more calorie expenditure. In addition, MyBehaviorCBP provides recommendations such that the total number of recommended minutes to exercise does not exceed 60 minutes. MyBehavior did not have any limit on total calories for the recommendations.

Despite the similarities between the two systems, the effect sizes in Table 2 are lower than those in the previous MyBehavior trials [36]. This is expected, since MyBehavior was designed for weight loss and did not deal with the negative psychological challenges in chronic pain. Indeed, out of the 321 reported daily surveys for the MyBehaviorCBP, in 39.9% cases negative emotions were reported in the PAM [57]. In a study with MyBehavior, out of 687 daily survey responses, 32.1% cases negative emotions were reported. Therefore, MyBehaviorCBP likely made participants active despite the prevalence of negative emotions.

### Relationship With Prior mHealth Apps for Chronic Pain Self-Management

Over the years, a variety of mobile apps have been proposed for chronic pain self-management, with some apps aiming at prescribing cognitive behavioral components [15,30,32]. Other apps use diary-based approaches by logging subjective self-reports and provide basic feedback such as reminders for medication [12], and some newer apps such as the WebMD PainCoach [31] enable pain monitoring, setting and tracking activity goals, and generating related messages. However, MyBehaviorCBP differs in that it uses an in-phone machine learning approach directly on tracked physical activity data and automatically generates new person-specific suggestions based entirely on this. This also lends MyBehaviorCBP’s automated approach to be complementary to other approaches (eg, methods based on cognitive behavioral therapy that could be combined with the tracked data-driven suggestions from MyBehaviorCBP).

### Limitations

One limitation is the small number of study participants and relatively short study length. However, MyBehaviorCBP is an early stage technology. It is difficult to acquire resources to conduct efficacy trials with unproven technology on a potentially vulnerable chronic pain population. As a result, the purpose of this pilot study was to inform feasibility and acceptability. In our future work, we will use the lessons learned in this pilot study to conduct longer term studies on a larger population and also to include specific back pain outcomes such as the Oswestry Low Back Pain Disability Questionnaire [59].

Another limitation is that MyBehaviorCBP does not fully address the question of whether even moderate exercise can have adverse consequences. If there is a short-term pain flare, then moderate exercising can temporarily increase pain and MyBehaviorCBP should not recommend exercising during a pain flare. However, it is not clear whether exercising has any long-term adverse effect on pain. Some prior work [60,61] argues exercising may have longer term effects and suggests activity pacing, where a participant engages in an active phase of exercise for a certain amount of days followed by a resting phase with decreased or no exercise. However, we are not aware of any published studies that have examined the question of long-term adverse effects empirically. Some providers recommend rest while others recommend that patients remain active without “overdoing it” based on their clinical experience. Future research is needed to address this important question of longer term effects. Using apps like MyBehaviorCBP could help address this question. For example, the static upper limit of a 60 minutes threshold could be dynamic. This value could be determined either automatically by a model-based approach or by an expert such as a physiotherapist. Should it be deemed that the user needs rest, the limit could be set to zero or a low value.

### Conclusion

In this paper, we presented the acceptability and feasibility of MyBehaviorCBP, a data-driven physical activity recommender system for chronic pain. We found preliminary evidence of increased walking activity; a few key areas of improvements have been also identified. In future work, we will incorporate these improvements and run a randomized controlled trial. If efficacy is demonstrated, then a technology like MyBehaviorCBP could have great promise because it is an automated system with no second person involved (eg, a physiotherapist). Also, all the data processing of MyBehaviorCBP is kept inside the phone which allows the app to preserve user privacy. Such automated and privacy-preserving features imply that MyBehaviorCBP has few barriers to scalability. 

## Abbreviations

AIC: Akaike information criterion
BIC: Bayesian information criterion
LR: likelihood ratio
MAB: multi-armed bandit
MET: metabolic equivalent of task
mHealth: mobile health
PAM: Photographic Affect Meter
RL: reinforcement learning
